# Case report of *Plasmodium ovale curtisi* malaria in Sri Lanka: relevance for the maintenance of elimination status

**DOI:** 10.1186/s12879-017-2411-z

**Published:** 2017-04-24

**Authors:** Sharmini Gunawardena, Rachel F. Daniels, Thishan C. Yahathugoda, Mirani V. Weerasooriya, Katelyn Durfee, Sarah K. Volkman, Dyann F. Wirth, Nadira D. Karunaweera

**Affiliations:** 1 Department of Parasitology, Faculty of Medicine, University of Colombo, 25, Kynsey Road, Colombo 8, Sri Lanka; 2 Department of Immunology and Infectious Diseases, Harvard T.H. Chan School of Public Health, Boston, MA USA; 3 Infectious Disease Initiative, The Broad Institute of MIT and Harvard, Cambridge, MA USA; 4 Department of Parasitology, Faculty of Medicine, University of Ruhuna, Karapitiya, Galle, Sri Lanka; 5 School of Nursing and Health Science, Simmons College, Boston, MA USA

**Keywords:** *Plasmodium ovale curtisi*, Elimination program, Malaria, Sri Lanka

## Abstract

**Background:**

Following its recent certification as malaria-free, imported infections now pose the greatest threat for maintaining this status in Sri Lanka. Imported infections may also introduce species that are uncommon or not previously endemic to these areas. We highlight in this case report the increasing importance of less common malaria species such as *Plasmodium ovale* in elimination settings and discuss its relevance for the risk of malaria resurgence in the country.

**Case presentation:**

A 41-year-old patient from southern Sri Lanka was diagnosed with malaria after 8 days of fever. Microscopy of blood smears revealed parasites morphologically similar to *P. vivax* and the rapid diagnostic test was indicative of non-*P. falciparum* malaria. He was treated with chloroquine over 3 days and primaquine for 14 days. He was negative for malaria at a one-year follow-up. Molecular testing performed subsequently confirmed that infection was caused by *P. ovale curtisi*. The patient gave a history of *P. vivax* malaria treated with chloroquine and primaquine. He also provided a history of travel to malaria endemic regions, including residing in Liberia from May 2012 to November 2013, throughout which he was on weekly malaria prophylaxis with mefloquine. He had also visited India on an eight-day Buddhist pilgrimage tour in September 2014 without malaria prophylaxis.

**Conclusions:**

It is crucial that every case of malaria is investigated thoroughly and necessary measures taken to prevent re-introduction of malaria. Accurate molecular diagnostic techniques need to be established in Sri Lanka for the screening and diagnosis of all species of human malaria infections, especially those that may occur with low parasitemia and are likely to be undetected using the standard techniques currently in use. In addition, ascertaining whether an infection occurred through local transmission or by importation is critical in the implementation of an effective plan of action in the country. This new era emphasizes the global nature of regional malaria elimination. Increasing global surveillance and tool development are necessary in order to “fingerprint” parasites and identify their origin.

## Background

Sri Lanka was recently certified as a nation free of malaria by the World Health Organization (WHO), having recorded its last indigenous case in October 2012 [1]. High vigilance has been maintained, with all reported cases of malaria being investigated either microscopically or by rapid diagnostic tests (RDT), with radical treatment and follow-up provided. Imported cases have posed the greatest threat for sustaining malaria elimination [2–4].

An indigenous case of *P. ovale* was reported from Sri Lanka in 2008 [5]. The patient had given a history of repeated attacks of *P. vivax* malaria, the last of which had occurred only two weeks prior to the episode of admission. Microscopy had revealed asexual stages resembling those of *P. vivax* and blood was sampled for the analysis of molecular markers of drug resistance. He had been treated as a case of treatment failure until molecular testing confirmed it as a *P. ovale* infection. This was considered an indigenous infection as the patient neither gave a history of travel overseas nor received any transfusion of blood or blood products [5].

Here we report another case of malaria due to *P. ovale* in Sri Lanka. Ascertaining whether this infection occurred through local transmission or by importation is critical in the implementation of an effective plan of action for malaria surveillance in Sri Lanka. The relevance of this case for the risk of malaria resurgence in the country is discussed.

## Case presentation

A 41-year-old police officer from Wanduramba, Galle (Southern Province, Sri Lanka) presented to the Teaching Hospital in Karapitiya on May 12, 2015, with fever, chills, and rigors that had persisted for three days. On examination, the patient was febrile but had no respiratory, gastro-intestinal, or circulatory abnormalities and no clinical evidence of any enlargement of the spleen or the liver. He had developed a fever while attending a residential meditation program at a center situated in Pinnawala, Rambukkana (Kegalle district, Sabaragamuwa Province), where he had been residing since the beginning of April 2015.

**Fig. 1:**
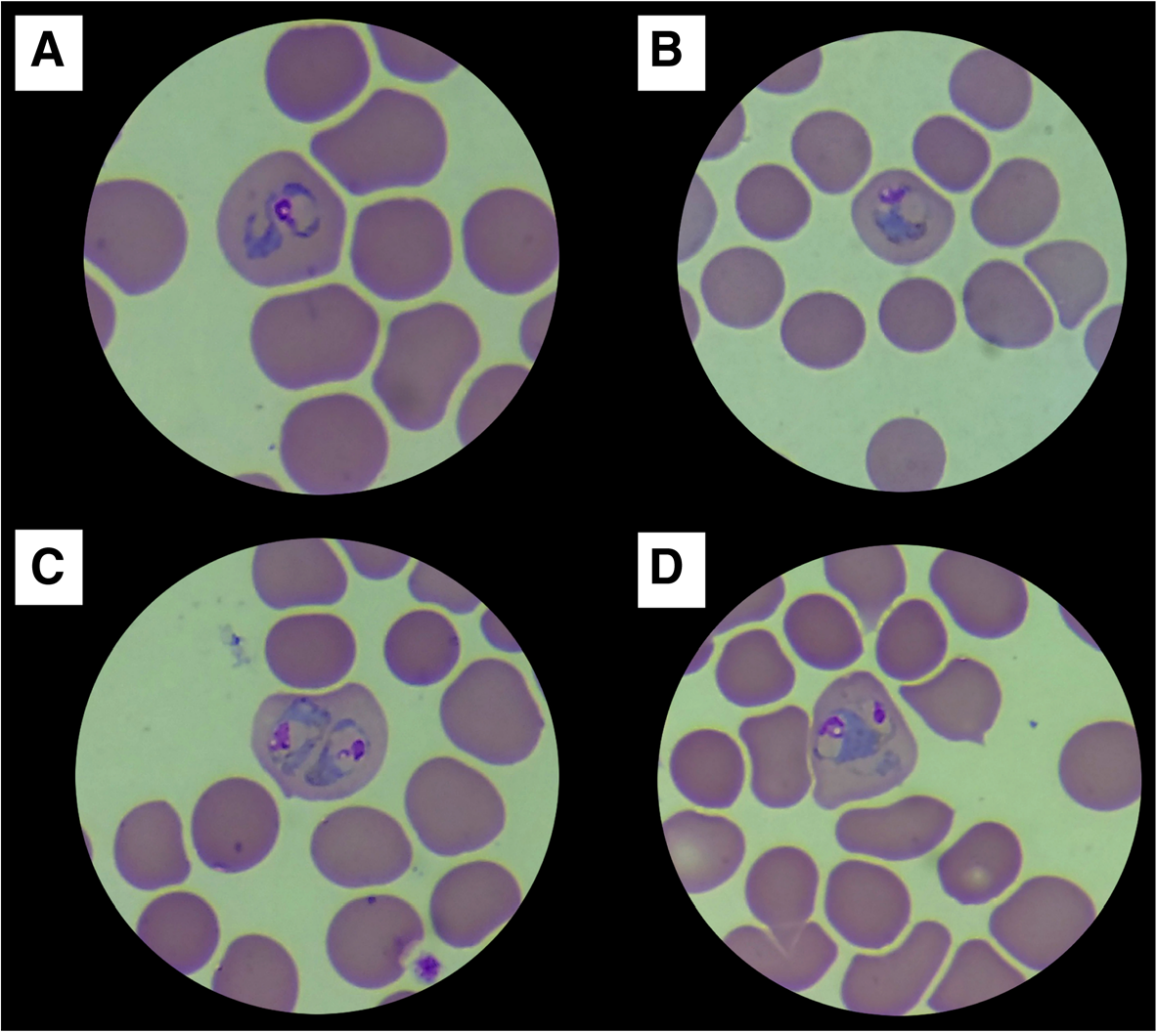
Giemsa stained thin blood smears (10 × 100 magnification). **a**-**d** Trophozoites of *Plasmodium ovale* Note the enlarged parasitized red cells (**a**-**d**) and the presence of multiple parasites within red cells (**c**, **d**).

He was positive for dengue antibodies and had a low platelet count (~77,000/μL; normal range: 150,000–400,000); based on these criteria, he was treated for dengue fever. Since the fever did not subside after eight days, the patient was referred to the Department of Parasitology at the Faculty of Medicine of the University of Ruhuna in order to exclude malaria. Microscopy of blood smears revealed parasites morphologically similar to *P. vivax* (Fig. 1), with a parasite count of 7695/μL and rapid diagnostic test (RDT, *CareStart* Malaria HRP2/pLDH Combo, Access Bio, Inc. USA) findings indicative of non-*P. falciparum* malaria. The patient was managed as a case of *P. vivax* malaria. He recovered completely following treatment with 25 milligram (mg) of chloroquine base per kilogram (kg) body weight (bw) administered over three days (10 mg, 10 mg, and 5 mg base per kg bw on days 1, 2, and 3 respectively) and was discharged with a 14-day course of primaquine at a daily dosage of 0·25 mg/kg bw to achieve a radical cure [6]. He was followed up by the Anti-Malaria Campaign (Ministry of Health, Sri Lanka) with weekly samples of blood tested for the presence of malaria antigens as well as smears evaluated for parasites during the initial 28 days after discharge, and continued monthly thereafter up to one year, with all antigen and blood smears negative for malaria.

Molecular testing was performed to confirm the diagnosis. However, it was not done for confirmation of parasite clearance at the end of follow up. Parasite DNA was extracted from a sample of venous blood collected prior to the commencement of treatment using a QIAamp DNA Blood Mini Kit (Qiagen GmbH, Hilden, Germany). To identify the species of *Plasmodium* present in the sample, the extracted DNA was first amplified by nested polymerase chain reaction (PCR) using the UNR and PLF and NewRevshort and NewPLFshort primers as previously described [7]. The nested PCR products were visualized by agarose gel electrophoresis and the bands compared to *P. falciparum*, *P. ovale*, and *P. malariae* positive controls from Senegal [8] as well as to a no-template water negative control. The positive colonies were cloned using a commercial kit (CloneJET PCR Cloning Kit, Thermo Scientific) following manufacturer directions and sent for Sanger sequencing (Macrogen Corp., Rockville, MD USA). A BLAST search of the NCBI database revealed that, contrary to the clinical suspicion, the patient sample was positive not for *P. vivax*, but for *P. ovale curtisi*, with a 99% identity to previously reported sequences. The patient sample sequence has been deposited to GenBank as accession number KY111277.

**Fig. 2:**
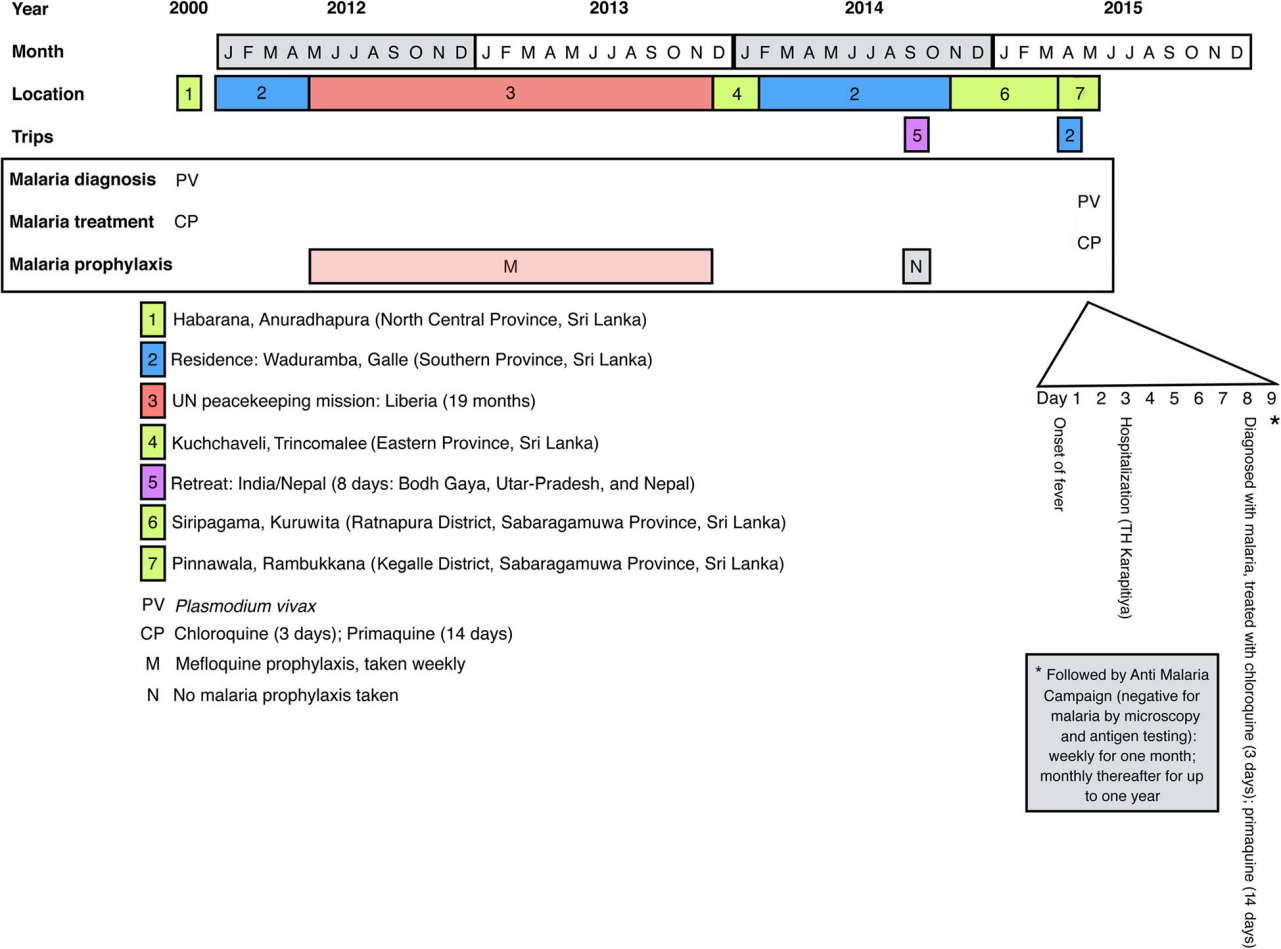
Timeline of events

To assess the possible sources of this malaria infection, a detailed history was taken (Fig. 2). The patient had a history of malaria contracted in 2000, while he was working in Habarana (Anuradhapura district, North Central Province). According to the information provided, his episode of *P. vivax* malaria was treated with three days of chloroquine and 14 days of primaquine. He also provided a history of travel to malaria endemic regions, including residing in Liberia from May 2012 to November 2013 as part of a United Nations peacekeeping mission. Throughout his stay in Liberia he was on weekly malaria prophylaxis with mefloquine. Upon returning to Sri Lanka, he was posted to Kuchchaveli, Trincomalee (Eastern Province, Sri Lanka), where he remained for two months. Thereafter, he had been living at home in Waduramba, Galle. His travel history also indicated he had visited India on an eight-day Buddhist pilgrimage tour in September 2014, when he stayed in Bodh Gaya, Uttar-Pradesh and Nepal during this visit. Malaria prophylaxis was not used during this tour.

From November 2014 to March 2015, he had been at a residential meditation center in Siripagama, Kuruwita (Ratnapura district, Sabaragamuwa Province). Thereafter, he had briefly visited his home in Galle. In early April 2015 he had gone to the meditation center in Pinnawala, Rambukkana, where he developed the current illness (Fig. 2).

The districts of Kegalle and Ratnapura, where the two meditation centers are located, are low endemic areas for malaria [1]. The meditation center in Siripagama is a popular retreat isolated from human habitation and surrounded by natural jungle, while the center at Pinnawala is more urbanized. At the time the patient was residing at these meditation centers, he had not encountered any foreign residents from whom the infection could have been transmitted.

## Discussion and Conclusions

In the context of the recent WHO certification of Sri Lanka as malaria-free [9], this case report is of particular interest, as it is a parable for the success and risks for countries nearing and maintaining malaria elimination status. Increased international travel correspondingly increases the risk of parasite transmission between and among malaria endemic and non-endemic nations, making the traditional paradigm of a geographically defensible country for malaria elimination no longer feasible. In countries with low transmission or that are maintaining their malaria-free status such as Sri Lanka, imported cases are of concern [10, 11]. These imported infections may introduce species that are uncommon or not previously endemic to these areas, and detection and diagnosis may be difficult due to declining microscopist accuracy due to lack of practice. *P. falciparum* and *P. vivax* are the most commonly reported species in Sri Lanka; thus, even trained microscopists may be challenged to accurately identify other *Plasmodium* species.

*P. ovale* is one of five species that cause human malaria infection, and is now known to be endemic across tropical regions in Africa and Asia [12–17]. Recent molecular studies indicate that *P. ovale* malaria is caused by two closely related species namely *P. ovale curtisi* and *P. ovale wallikeri* [18, 19]. Diagnosis of *P. ovale* malaria can be difficult because of low parasitemia levels, mixed infections with other *Plasmodium* species, and false negative results from malaria RDTs [20]. The developmental cycle of *P. ovale* is very similar to that of *P. vivax,* including changes produced in the infected erythrocytes, and giving cause to tertian fever as well as generating relapses from latent parasites in the liver [13]. While *P. ovale* has traditionally been considered to cause benign clinical disease, several cases with severe complications have been reported [21, 22]. The reported relapse interval after treatment of a primary attack for *P. ovale* ranges from 17 to 255 days [13]. However, delayed primary attacks with *P. ovale* have been observed even after 4 years [23, 24].

The similarity of *P. ovale* parasites to *P. vivax* microscopically in blood smears as well as in its clinical course and response to treatment could easily lead to misdiagnosis based on microscopy or RDTs. In this instance, the presence of multiple parasites within red cells as seen in the blood smears led to further investigation based on molecular techniques which helped to differentiate between *P. ovale* and *P. vivax* and further define the infection as *P. ovale curtisi*. Globally, molecular testing in Africa and other Asian settings has revealed a higher incidence of these infections than has been previously reported, and the increasing importance of *P. ovale* and *P. malariae* in a malaria elimination setting in China was recently highlighted [25–27].

Furthermore, the potential introduction of new or uncommon species increases concerns regarding the vector population. The natural vectors of *P. ovale* in Africa are believed to be *Anopheles gambiae* and *A. funestus*, while a few other species, namely *A. albimanus*, *A. quadrimaculatus*, *A. freeborni*, *A. maculatus*, *A. subpictus*, *A. stephensi* and *A. dirus,* have the potential for transmission [13]. *Anopheles culicifacies* is the major vector of malaria transmission in Sri Lanka, while *A. subpictus* is a secondary vector [1]. The ability of *A. culicifacies* to transmit *P. ovale* is not known. However, *A. subpictus* as well as *A. maculatus,* which are considered potential vectors of *P. ovale*, are endemic in Sri Lanka [28, 29]. Thus, early detection and treatment of all imported cases becomes imperative in the presence of mosquito vectors and other environmental factors that remain conducive for infection transmission.

At this juncture, it is crucial that every case of malaria is investigated thoroughly and necessary measures taken to prevent the re-introduction of malaria. It is doubly so in this case, as *P. ovale* is not considered endemic in Sri Lanka and thus determining the parasite origin of importation would be of utmost importance for ongoing surveillance efforts. This patient had traveled widely, visiting malaria endemic areas both within and beyond the shores of the country. During his stay in Liberia 1.5 years prior to his illness, he had been on malaria prophylaxis with mefloquine. Suppressive prophylaxis provided by mefloquine is effective against *P. falciparum,* but will not prevent relapses caused by *P. ovale* [30]. Thus, a *P. ovale* infection acquired while residing in Liberia could have relapsed several years later. Sporadic cases of *P. ovale* malaria have also been reported from various parts of India [31, 32]. Since transmission of *P. ovale* in India is not widespread, it seems likely that this patient acquired his infection from Liberia where *P. ovale* is known to be endemic [12, 13].

This new era emphasizes the global nature of regional malaria elimination. Now more than ever is there an urgent need for next-generation detection methods, not just for the species historically present in the population, but also for introduced or uncommon species. Accurate molecular diagnostic techniques need to be established in Sri Lanka for the screening and diagnosis of all species of human malaria infections, especially those that may occur with low parasitemia and likely to be undetected by the routine techniques currently in use. In this case, although the detailed history was suggestive of an imported transmission, confirmation of parasite origin was not possible. Increasing global surveillance and tool development is required to “fingerprint” parasites and identify their origin. The next generation of ‘network communities’ utilizing genomic advances may offer increased ability to identify ‘hotspots’ and ‘hot-pops’, the source and sink of parasites, on a global scale [33].

## Abbreviations

BLAST: Basic Local Alignment Search Tool
bw: Body weight
kg: Kilogram
mg: Milligram
NCBI: National Center for Biotechnology Information
PCR: Polymerase chain reaction
RDT: Rapid diagnostic tests
WHO: World Health Organization
